# Focus Group Evaluation from the Perspective of Program Implementers: Findings Based on the Secondary 2 Program

**DOI:** 10.1100/tsw.2009.117

**Published:** 2009-10-01

**Authors:** Daniel T. L. Shek, Rachel C. F. Sun, Christina Y. P. Tang

**Affiliations:** ^1^ Department of Applied Social Sciences, The Hong Kong Polytechnic University, Hong Kong, China; ^2^ Department of Sociology, East China Normal University, Shanghai, China; ^3^ Kiang Wu Nursing College of Macau, Macau, China; ^4^ Department of Social Work, Chinese University of Hong Kong, Hong Kong, China

**Keywords:** positive youth development program, Hong Kong, qualitative evaluation, focus group

## Abstract

Nine focus groups comprising 23 program implementers recruited from nine schools were conducted to evaluate the Tier 1 Program (Secondary 2 Program) of the Project P.A.T.H.S. (Positive Adolescent Training through Holistic Social Programmes). Qualitative findings showed that a majority of the program implementers regarded the program as beneficial to the program participants in different psychosocial domains. The program implementers also described the program positively and positive metaphors were used to represent the program. In conjunction with the previous research findings, the present study provides further support for the effectiveness of the Tier 1 Program of Project P.A.T.H.S. in promoting holistic development among Chinese adolescents in Hong Kong.

## INTRODUCTION

To promote holistic development among adolescents in Hong Kong, The Hong Kong Jockey Club Charities Trust approved HK$400 million to launch a project entitled "P.A.T.H.S. to Adulthood: A Jockey Club Youth Enhancement Scheme". The acronym "P.A.T.H.S." denotes **P**ositive **A**dolescent **T**raining through **H**olistic **S**ocial Programmes. There are two tiers of program (Tier 1 and Tier 2) in this project. The Tier 1 Program is a universal positive youth development program for students from Secondary 1 to 3, using a set of multiyear curricula developed by the research team comprising scholars from different disciplines, including social work, psychology, education, and rehabilitation and health. The Tier 2 Program is a selective program provided for students with greater psychological needs at each grade.

As an exemplary youth development program emphasizing the importance of evidence-based practice, the Tier 1 Program of the Project P.A.T.H.S. was systematically designed based on sound theories and research findings[1,2,3]. Moreover, rigorous evaluation on the effectiveness of the Tier 1

Program has also been carried out, with triangulation by different data sources (e.g., program implementers and participants), different methods (objective outcome evaluation, subjective outcome evaluation, qualitative evaluation, and process evaluation), researchers (e.g., intra- and inter-rater reliability checking), and data types (quantitative data and qualitative data).

There are several lines of evidence supporting the effectiveness of the Tier 1 Program. First, objective outcome evaluation at pre- and post-test[4,5], as well as subjective outcome evaluation collected from the program participants and implementers[6,7,8,9,10,11,12], showed that the program was beneficial to the participants. Second, qualitative evaluation based on focus group interviews[13,14,15,16], analyses of students' weekly diaries[17], and case studies[18,19] also showed that both program participants and implementers had positive perceptions of the program and regarded the program as helpful to the students' overall development. Third, process evaluation based on systematic classroom observation[20,21] and interim evaluation[22,23] showed that the implementation fidelity was high and the program was well received, although some implementation difficulties were also reported.

Morgan[24] pointed out that the focus group interview is a good strategy among various qualitative evaluation strategies because it produces an opportunity to collect data from a group that is discussing topics of interest to the researchers. It also combines the advantages of both individual interview and participant observation. First, as the researcher defines the discussion topics, the focus group is less spontaneous than participant observation. Second, because of the participant-defined nature of group interaction, the focus group setting is more flexible than individual interview. Furthermore, the focus group allows generation of rich information via respondents' interactions, which can hardly be obtained in individual interview[25,26]. In addition, the information collected could be ecologically valid if the focus group is conducted in corresponding social setting[27].

As qualitative findings can give an in-depth picture on the effects of a program, the present study was carried out to evaluate the Tier 1 Program of the Project P.A.T.H.S. based on qualitative findings generated from focus group interviews conducted in secondary schools. Although positive findings were found in the previous studies based on focus group interviews, the findings were limited to the Secondary 1 level in the Experimental Implementation Phase (2005/06 academic year)[13,14] and the Full Implementation Phase (2006/07 academic year)[15,16]. Therefore, it is important to examine the perceptions of the program implementers when they implemented the Secondary 2 Program.

The present study attempted to examine the program implementers' views on the Tier 1 Program "because personnel, consciously or unconsciously, influence the effectiveness of prevention program lessons, it is important to assess their perceptions when evaluating a specific program to provide insight into the context in which the program operates" ([28], p. 219). Furthermore, several arguments can be put forward to support the importance of evaluating youth development programs from the instructors' perspective. First, in the utilization-focused evaluation paradigm, the Joint Committee on Standards for Educational Evaluation[29] indicates that different stakeholders should be identified (Standard U1) and their views should be taken into account (Standard F2). Second, as program implementers are usually more experienced than the students, it can be argued that the implementers have better skills and experiences in judging the quality of the program designed; hence, they are in a better position to assess the program effectiveness. Third, in the Project P.A.T.H.S., although teachers and social workers are expected to carry out both program implementation and evaluation tasks in their practice, role conflict is not a problem because they are basically trained to carry out reflective practice and these professions actually encourage professionals to evaluate the delivered programs in an honest and sincere manner.

The purpose of this paper is to evaluate the effectiveness of the Project P.A.T.H.S. via qualitative evaluation based on focus group interviews with program implementers delivering the Tier 1 Program (Secondary 2 Program) in the Full Implementation Phase in the 2007/08 academic year. A general qualitative orientation was adopted in this study, in which general strategies of qualitative research are employed (e.g., collection of qualitative data, respecting the views of the informants, data analysis without preset coding scheme), but a specific qualitative approach is not adhered to. According to Shek et al.[30], the general qualitative orientation is the most commonly used qualitative research approach in field of Social Work.

## METHODS

### Participants

Among the schools that joined the Secondary 2 Program of the Project P.A.T.H.S. in the Full Implementation Phase in the 2007/08 academic year (n = 196), there were 80 schools that adopted the full program (i.e., 20-h program involving 40 units) and 116 schools that adopted the 10-h core program. In the sampling process, eight randomly selected schools that joined the full program and two randomly selected schools that joined the core program were invited to participate in the focus group interviews. However, a school that adopted the 20-h program declined because its instructors were busy at term end and could not schedule a time slot for interviews. Among the nine schools, eight schools integrated the Tier 1 Program into their formal curriculum (e.g., Life Education and Civic Education), while one school implemented the Tier 1 Program in form teacher lessons. The average number of classes per school was 4.9 (range: 3–6). The program implementers in these nine schools were invited to join the focus group interviews. With the school as a unit, nine focus groups were formed, comprising 15 teachers and eight social workers. The average number of respondents per group was 2.6 (range: 1–5).

### Procedures

Informants' consent was obtained prior to the study. All focus group interviews were jointly conducted by two trained colleagues. There were four trained interviewers in total, one of whom had a doctoral degree and the others were registered social workers. During the interviews, the respondents were encouraged to verbalize their views and perceptions of the program. With respect to Principle 3 (i.e., detailed description of the data collection procedures) suggested by Shek et al.[30], the broad interview guide of the focus group interviews conducted is presented in Table 1. The interview questions were designed with reference to the CIPP model[31] and previous research[32]. In the interviews, the interviewers adopted the role of facilitators, and were conscious of being open to accommodate both positive and negative experiences expressed by the informants. As the interviewers either had training in social group work and/or substantial group work experience, they were conscious of the importance of encouraging the informants to express views of a different nature, including both positive and negative views.

### Data Analyses

The interviews were audio recorded, with the respondents' consent. The content of the interviews was fully transcribed by student helpers and checked for accuracy by two other trained helpers. In the coding process, another two research assistants, who were not involved in the data collection, were involved in the data analyses of the narratives. The unit of analysis was a meaningful unit instead of a statement. For example, the statement that a program was "worthy and amusing" was broken down into two meaningful units or attributes, namely, "worthy" and "amusing". On the other hand, descriptions with similar meaning (e.g., "good quality" and "high quality") were grouped into the same attribute category. The present coding system was developed after much consideration of the raw data and several preliminary analyses.

After initial coding, the positivity nature of the codes was determined, with four possibilities (positive code, negative code, neutral code, and undecided code). To enhance the reliability of the coding on the positivity nature of the raw codes, both intra- and inter-rater reliability were carried out. Because of space limitation, qualitative findings on three areas are presented in this paper: (1) descriptors that were used by the informants to describe the program, (2) metaphors (i.e., incidents, objects, or feelings) that were used by the informants to stand for the program, and (3) implementers' perceptions of the benefits of the program to students.

## TABLE 1
## Interview Guide for the Focus Group Interviews Involving the Implementers

**A. Context Evaluation:**
- How much do you know about "Positive Youth Development Programs" (e.g., "Life Skills Education")? What is your overall impression of these programs?
- Have you taught programs that are similar to the Project P.A.T.H.S. before?
- If yes, how effective do you feel they are?
- From your perspective, what are the differences between the Project P.A.T.H.S. and other similar programs?
- Do you agree with the vision of the Project P.A.T.H.S.? Why?

**B. Input Evaluation:**
- What kind of effects do you feel that the implementation of the Project P.A.T.H.S. have on the school's normal operation?
- If the school incorporates the Project P.A.T.H.S. curriculum into the normal curriculum (e.g., Life Education, Integrated Humanities, etc.), from your perspective, what are the advantages and disadvantages of this arrangement?
- If the school does not incorporate the Project P.A.T.H.S. curriculum into the normal curriculum (e.g., homeroom, extracurricular activities, etc.), do you feel that this arrangement is successful?
- To accommodate the implementation of the Project P.A.T.H.S., did the school make special arrangements?
- Do you feel that the principal and administrative staff support the implementation of the Project P.A.T.H.S. at your school? Why or why not?
- Do you feel that the training you received is adequate for you to carry out the program requirements?

**C. Process Evaluation:**

**1. General Impression of the Program**
- What is your overall impression of the program? What are your feelings?
- All in all, did you enjoy leading the program?
- Regarding the program, what has given you a lasting impression?
- While implementing the program, did you have any unforgettable experiences?

**2. Comments on the Program Content**
- Regarding the program, what are the things you like? And what are the things you dislike?
- What are your views on the different units and content of the program?
- Which units do you like the most? Why?
- From your recollection, are there any activities that aroused students' interest to participate in the program?

**3. Comments on the Program Implementation**
- While implementing the program, did you encounter any difficulties?
- Do you feel that the program implementation was successful?
- To what degree/extent did you follow the program curriculum manuals? Why?
- What are your thoughts on the students' responses to the program?

**D. Product Evaluation**

**1. Evaluation of the General Effectiveness of the Program**
- Do you feel that the program is beneficial to the development of adolescents?
- Have you noticed any changes in students after their participation in the program? If yes, what are the changes? (free elicitation)
- If you noticed changes in students, what do you think are the factors that caused such changes?
- If you have not noticed changes in students, what do you think are the factors that caused students not to change?

**2. Evaluation of the Specific Effectiveness of the Program**
- Do you think that the program can promote students' self-confidence/ability to face the future?
- Do you think that the program can enhance students' abilities in different areas?

Optional Questions:
- Do you think that the program can enhance students' spirituality aspect?
- Do you think that the program can promote students' bonding with family, teachers, and friends?
- Do you think that the program can establish students' compassion and care for others?
- Do you think that the program can promote students' participation and care for society?
- Do you think that the program can promote students' sense of responsibility to the society, family, teachers, and peers?

**3. The Program's Impact on the Instructor**
- Do you feel you have gained something by leading this program? And have you lost something?
- If you have the opportunity in future, do you wish to lead similar programs again?

**4. Other Comments**
- If you are invited to use three descriptive words to describe the program, what are the three words that you would use?
- If you are invited to use one incident, object/thing, or feeling (e.g., indigestion, enjoyment, child at heart, etc.) to describe the program, how would you describe the program?

### Ideological Biases and Preoccupations as Well as Strategies to Deal with Them

Shek et al.[30] argued for the importance of discussing the ideological biases and preoccupations of the researchers in a qualitative evaluation report (Principle 4). As program developers, the authors might have the preoccupation that the implemented program was good and it was beneficial to the students. In addition, the researchers might have the tendency to look at positive evidence rather than negative evidence. Thus, several safeguards against the subtle influence of such ideological biases and preoccupations were included in the present study (Principle 5). First, the researchers were conscious of the existence of ideological preoccupations (e.g., positive youth development programs are beneficial to adolescents) and carried out data collection and analyses in a disciplined manner. Second, both inter- and intrarater reliability checks on the coding were carried out (Principle 6). Third, multiple researchers and research assistants were involved in the data collection and analysis processes (Principle 7). Fourth, the first author was conscious of the importance and development of audit trails (Principle 9). The audio files, transcriptions, and steps involved in the development of the coding system were properly documented and systematically organized.

## RESULTS

For the descriptors used by the informants to describe the program, there were 44 raw descriptors and they could be further categorized into 33 categories (Table 2). Among these descriptors, 36 (81.8%) of them were coded as positive descriptors. For instance, *"Continuous effort, up-to-date information, sharing"* (Instructor A in School A) were the three descriptors given by one of the instructors to depict the program. In order to examine the intrarater reliability of the coding, each of the two research assistants recoded 20 randomly selected raw descriptors (without knowing the original codes given) at the end of the scoring process, and the intrarater agreement percentages calculated on the positivity of the coding for these descriptors were 100 and 100%, respectively. Also, the same 20 randomly selected descriptors were coded by another two colleagues with Master's degrees, without knowing the original codes given, with each of them coding 20 descriptors. The inter-rater agreement percentages calculated on the positivity of the coding were 85 and 100%, respectively.

As shown in Table 3, 14 metaphors were used by the informants to stand for the program and nine of them (64.3%) were regarded as positive metaphors, while five were regarded as neutral metaphors. Reliability tests showed that the intrarater agreement percentages calculated on the positivity of the coding for these metaphors were 100 and 100%, respectively, by the two research assistants. Similarly, the inter-rater agreement percentages calculated on the positivity of the coding were 100 and 100%, respectively, by another two colleagues with Master's degrees. One of the positive metaphors and one of the neutral metaphors used for portraying the program are shown below.

> *"Firework, very resplendent. That is, many unexpected things would happen…and it is very excited every time… Therefore, I think, first, there are surprises; second, there is much interaction, which in fact is enjoyable…"* (Instructor B in School B)

> *"Retractable telescope. We cannot see the (program) effect immediately, because we like to invite students to have long-term views. However, if the students do not know how to use (the retractable telescope), they may see some inverse image, or even see nothing else. In fact, we hope to teach them how to use in the process, and how to see a bit farther, wishing (one) can see beautiful things."* (Instructor C in School C)

Regarding the perceived benefits of the program to the program participants, 120 responses were recorded involving 29 attributes categorized into benefits at general level, interpersonal level, personal level, and others (Table 4). The findings showed that 106 responses (88.3%) were coded as positive responses. An example was shown in the following narratives of an instructor.

**TABLE 2**
**Descriptors Used by the Implementers to Describe the Program**

| **Responses** | **Nature of the Response** | | | | **Total** |
|---|---|---|---|---|---|
| | **Positive** | **Neutral** | **Negative** | **Undecided** | |
| Comprehensive | 1 | | | | 1 |
| Systematic | 1 | | | | 1 |
| Diversified | 2 | | | | 2 |
| Cognitive enhancement | 1 | | | | 1 |
| Fruitful | 4 | | | | 4 |
| Sometimes touching | 1 | | | | 1 |
| Match the topic very much | 1 | | | | 1 |
| (Very) Positive | 3 | | | | 3 |
| Interesting | 4 | | | | 4 |
| Effective | 1 | | | | 1 |
| Step by step | 1 | | | | 1 |
| Rare | 1 | | | | 1 |
| Interactive | 1 | | | | 1 |
| Excited | 1 | | | | 1 |
| Good feelings | 1 | | | | 1 |
| Very practical | 1 | | | | 1 |
| Very relaxed | 1 | | | | 1 |
| Meaningful | 1 | | | | 1 |
| Worthy to implement | 1 | | | | 1 |
| Closely connected with life | 1 | | | | 1 |
| Satisfied | 1 | | | | 1 |
| Glad | 1 | | | | 1 |
| Have gains | 1 | | | | 1 |
| Have positive expectation | 1 | | | | 1 |
| Hardworking | 1 | | | | 1 |
| Up-to-date information | 1 | | | | 1 |
| Sharing | 1 | | | | 1 |
| Bittersweet | | 1 | | | 1 |
| Partially uncertain | | 1 | | | 1 |
| Too wide (scope) | | | 1 | | 1 |
| Lack of time | | | 3 | | 3 |
| Worried | | | 1 | | 1 |
| Exclamation mark | | | | 1 | 1 |
| **Total responses** | **36** | **2** | **5** | **1** | **44** |

*"If s/he (the student) can point out one of the memorable things, or some activities s/he still have impression in mind, no matter games, instructors' words, or a moment, I think it is already a benefit in his/her life. That is, it is good actually. That is, s/he receives some messages first. In fact, how the future will be is depending on him/her, in his/her heart, it blossoms, or can be applied to daily life."* (Instructor D in School D)

**TABLE 3**
**Metaphors Used by the Implementers to Represent the Program**

| Metaphors | Nature of the Response | | | | Total |
|---|---|---|---|---|---|
| | Positive | Neutral | Negative | Undecided | |
| Perceiving the elephant in blind | | 1 | | | 1 |
| Firework | 1 | | | | 1 |
| Buffet | 1 | | | | 1 |
| Comb | 1 | | | | 1 |
| Gift | 1 | | | | 1 |
| Durian | | 1 | | | 1 |
| Clock | | 1 | | | 1 |
| Baby bird | 1 | | | | 1 |
| Wikipedia | 1 | | | | 1 |
| New product | | 1 | | | 1 |
| Retractable telescope | | 1 | | | 1 |
| Seeding | 1 | | | | 1 |
| Growing flower | 1 | | | | 1 |
| Sunshine | 1 | | | | 1 |
| **Total responses** | **9** | **5** | **0** | **0** | **14** |

Reliability tests showed that the intrarater agreement percentages calculated on the positivity of the coding from these perceived program benefits were 100 and 95%, respectively, by the two research assistants, whereas the inter-rater agreement percentages calculated on the positivity of the coding were 95 and 85%, respectively, by another two colleagues with Master's degrees.

## DISCUSSION

The aim of the present study was to examine the effectiveness of the Tier 1 Program based on the qualitative evaluation of the focus group interviews with program implementers. Two major conclusions can be drawn from this study. First, the program was basically perceived in a positive manner from the perspective of the program implementers (Tables 2 and 3). Although some teachers and social workers perceived the program in a negative manner (e.g., lack of time), this was not the dominant view. In fact, comparatively more implementers perceived the program to be fruitful and interesting. The findings based on the metaphors also showed that most of the responses were positive in nature, although the percentage of "neutral" responses was quite substantial. The present findings are generally consistent with those reported previously[16], suggesting that the program implementers perceived the Tier 1 Program in a positive manner.

Second, nearly fourth-fifths of the implementers perceived the program to be beneficial to students (Table 4). On the personal level, many implementers reported that this program had strengthened the students' behavioral competence (e.g., students exhibited more positive behaviors) and personal growth (e.g., enhanced abilities and willingness to express themselves). On the interpersonal level, many instructors thought that this program not only strengthened students' social skills, but also enhanced student-instructor relationships. These positive findings are consistent with previous findings obtained in the Secondary 1 Program[13,14,15,16]. Following the principle of triangulation, the present findings, based on the implementers' perspective, echoed with the previous quantitative and qualitative evaluation findings that the Tier 1 Program was effective in promoting the holistic development among the program participants.

**TABLE 4**
**Benefits of the Tier 1 Program Perceived by the Implementers**

| Category | Subcategory | Responses | Nature of the Response | | | | Total |
|---|---|---|---|---|---|---|---|
| | | | Positive | Neutral | Negative | Undecided | |
| General | | Have significant positive influences on students | 7 | | | | 7 |
| | | Have some kind of help to students | 16 | | | | 16 |
| | | Its effectiveness will be shown in the long run | 7 | | | | 7 |
| | | Unable to observe the changes within a short period of time | | | | 3 | 3 |
| | | Students are unable to perceive immediate changes in themselves | | | | 1 | 1 |
| | | Unable to notice the immediate changes in students' resilience | | | | 1 | 1 |
| | | Unable to notice the immediate changes in values and identity | | | | 2 | 2 |
| | | The effectiveness depends on the students' learning attitudes | | 2 | | | 2 |
| | | Difficult to measure the effectiveness | | | | 2 | 2 |
| | | Little enhancement on academics | 1 | | | | 1 |
| | | Unable to help the students with special needs | | | 1 | | 1 |
| | | **Subtotal** | **31** | **2** | **1** | **9** | **43** |
| Interpersonal Level | | Enhanced student-instructor relationships | 20 | | | | 20 |
| | | Enhanced peer relationship and cooperation | 6 | | | | 6 |
| | | Enhanced social skills | 9 | | | | 9 |
| | | **Subtotal** | **35** | **0** | **0** | **0** | **35** |
| Personal Level | Personal Growth | Increased students' ability and willingness to express themselves | 7 | | | | 7 |
| | | Increased maturity | 1 | | | | 1 |
| | | **Subtotal** | **8** | **0** | **0** | **0** | **8** |
| | Emotional Competence | Enhanced emotional management | 3 | | | | 3 |
| | | **Subtotal** | **3** | **0** | **0** | **0** | **3** |
| | Behavioral Competence | Enhanced problem-solving skills | 1 | | | | 1 |
| | | Enhanced conflict management | 1 | | | | 1 |
| | | Strengthened positive behaviors | 5 | | | | 5 |
| | | Application of learned things to daily life | 1 | | | | 1 |
| | | Learned to appreciate, take care of, and respect others | 3 | | | | 3 |
| | | Take initiative | 3 | | | | 3 |
| | | **Subtotal** | **14** | **0** | **0** | **0** | **14** |
| | Cognitive Competence | Enhanced critical thinking | 2 | | | | 2 |
| | | Enhanced self-reflection | 4 | | | | 4 |
| | | **Subtotal** | **6** | **0** | **0** | **0** | **6** |
| | Moral Competence | Enhanced moral competence | 2 | | | | 2 |
| | | **Subtotal** | **2** | **0** | **0** | **0** | **2** |
| Others | | Enhancing instructors' knowledge and development | 7 | | | | 7 |
| | | Promoting schools' concern on student development | 1 | | | | 1 |
| | | Others | | | | 1 | 1 |
| | | **Subtotal** | **8** | **0** | **0** | **1** | **9** |
| **Total responses** | | **Total** | **106** | **12** | **1** | **1** | **120** |

On the other hand, it is noteworthy that there was a negative view that the program was unable to help students with special needs (Table 4). However, it is also noteworthy that the Tier 1 Program of the Project P.A.T.H.S. is a universal program designed for all junior secondary school students, with students having unique psychosocial needs being invited to join the Tier 2 Program that is tailor-made for these students by the school social services agency. Therefore, students with special psychosocial needs can join the Tier 2 Program to receive appropriate service. Honestly, an effective program that can successfully promote positive changes in students requires collaboration among several stakeholders, and sufficient facilities and backup[19].

In addition, some of the implementers reported that they were unable to observe immediate positive changes in students. There are several factors contributing to this observation. First, since the students only attended 10 or 20 h of training per grade for 2 years only, it might not be lengthy enough for them to acquire changes. In other words, the dosage of the program may affect the outcome. Second, because the positive youth development program commonly adopts the experiential learning approach in which learning is acquired by participating, experiencing, and reflecting[33], changes in students may need consolidation and integration. Unlike academic learning that can be measured by simple knowledge acquisition, it is important to provide sufficient opportunities for students to experience, do experiments, reflect, and conceptualize. For instance, instructors can carry out some additional activities after each lesson, such as exercises in program handbooks, and encourage students to apply what they learned in lessons to daily life so as to consolidate students' learning. In sum, both in-depth and extensive exposure is pertinent in creating prominent positive changes in students.

According to Shek et al.[30], it is important to look at alternative explanations in the interpretations of qualitative evaluation findings (Principle 10). There are several alternative explanations of the present findings, but they can be partially dismissed. First, although the findings can be explained in terms of demand characteristics, this explanation was not likely because the implementers were encouraged to voice their views without restriction and negative voices were, in fact, heard. Second, although it can be argued that the favorable findings were due to ideological biases of the researchers, several safeguards (e.g., intra- and inter-rater reliability, disciplined data collection, analyses, and interpretations) were used to reduce biases in the data collection and analysis process. Finally, it may be argued that the perceived benefits were due to other youth enhancement programs. However, this argument can be partially dismissed as the implementers in the focus group interviews were specifically asked about the program effects of the Project P.A.T.H.S. only.

There are several limitations of the study that should be discussed[30]. First, although the number of schools and implementers participating in this study is respectable, it would be helpful if more schools and implementers stratified according to school types (e.g., different academic and socioeconomic background) could be recruited. Second, only one-shot interviews were conducted for each focus group, and thus it would be illuminating if regular and ongoing qualitative evaluation data could be collected in each school. Third, besides focus groups, in-depth individual interviews would enable the researchers to understand the inner worlds and subjective experiences of each program implementer. Finally, although nearly all principles proposed by Shek et al.[30] were upheld in this study, peer checking and member checking (Principle 8) were not carried out in this study because of time and manpower constraints. Despite these limitations, this study provides additional qualitative evaluation findings supporting the positive nature of the Project P.A.T.H.S. and its effectiveness in promoting holistic youth development among Chinese adolescents in Hong Kong.

## ACKNOWLEDGMENTS

The preparation for this paper and the Project P.A.T.H.S. were financially supported by The Hong Kong Jockey Club Charities Trust.

## References

1. Catalano, R.F., Berglund, M.L., Ryan, J.A.M., Lonczak, H.S., and Hawkins, J.D. (2002) Positive Youth Development in the United States: Research Findings on Evaluations of Positive Youth Development Programs. Available from http://aspe.hhs.gov/hsp/PositiveYouthDev99/

2. Shek, D.T.L. and Ma, H.K. (2006) Design of a positive youth development program in Hong Kong. *Int. J. Adolesc. Med. Health* **18(3)**, 315-327.
3. Ma, H.K. and Sun, R.C.F. (2006) Development of a positive youth development program in Hong Kong: overview of the proposed curriculum for the junior secondary school years. *Int. J. Adolesc. Med. Health* **18(3)**, 417–431.
4. Shek, D.T.L. (2009) Effectiveness of the Tier 1 Program of Project P.A.T.H.S.: findings based on the first 2 years of program implementation. *TheScientificWorldJOURNAL* **9**, 539–547.
5. Shek, D.T.L., Siu, A.M.H., Lee, T.Y., Cheung, C.K., and Chung, R. (2008) Effectiveness of the Tier 1 Program of Project P.A.T.H.S.: objective outcome evaluation based on a randomized group trial. *TheScientificWorldJOURNAL* **8**, 4–12.
6. Shek, D.T.L. and Ma, H.K. (2007) Subjective outcome evaluation of the Project P.A.T.H.S.: findings based on the program participants. *TheScientificWorldJOURNAL* **7**, 47–55.
7. Shek, D.T.L., Siu, A.M.H., and Lee, T.Y. (2007) Subjective outcome evaluation of the Project P.A.T.H.S.: findings based on the perspective of the program implementers. *TheScientificWorldJOURNAL* **7**, 195–203.
8. Shek, D.T.L. and Sun, R.C.F. (2007) Subjective outcome evaluation of the Project P.A.T.H.S.: qualitative findings based on the experiences of program implementers. *TheScientificWorldJOURNAL* **7**, 1024–1035.
9. Shek, D.T.L. and Sun, R.C.F. (2007) Subjective outcome evaluation of the Project P.A.T.H.S.: qualitative findings based on the experiences of program participants. *TheScientificWorldJOURNAL* **7**, 686–697.
10. Shek, D.T.L. (2008) Evaluation of the Tier 1 Program of Project P.A.T.H.S.: secondary data analyses of conclusions drawn by the program implementers. *TheScientificWorldJOURNAL* **8**, 22–34.
11. Shek, D.T.L., Sun, R.C.F., and Chan, C.W.Y. (2008) Evaluation of Project P.A.T.H.S. (Secondary 2 Program) by the program participants: findings based on the Experimental Implementation Phase. *TheScientificWorldJOURNAL* **8**, 526–535.
12. Shek, D.T.L., Sun, R.C.F., and Lung, D.W.M. (2008) Evaluation of Project P.A.T.H.S. (Secondary 2 Program) by the program implementers: findings based on the Experimental Implementation Phase. *TheScientificWorldJOURNAL* **8,** 536–546.
13. Shek, D.T.L., Lee, T.Y., Siu, A., and Lam, C.M. (2006) Qualitative evaluation of the Project P.A.T.H.S. based on the perceptions of the program participants. *TheScientificWorldJOURNAL* **6**, 2254–2263.
14. Shek, D.T.L. and Shik, A.W.Y. Qualitative evaluation of the Project P.A.T.H.S. in Hong Kong: findings based on the program implementers. *Int. J. Adolesc. Med. Health*, in press.
15. Shek, D.T.L. and Lee, T.Y. (2008) Qualitative evaluation of the Project P.A.T.H.S.: findings based on focus groups with student participants. *Int. J. Adolesc. Med. Health* **20(4)**, 449-462.
16. Shek, D.T.L. and Sun, R.C.F. Qualitative evaluation of the Project P.A.T.H.S. (Secondary 1 Program) based on the perceptions of the program implementers. *Int. Public Health J.*, in press.
17. Shek, D.T.L., Sun, R.C.F., Lam, C.M., Lung, D.W.M., and Lo, S.C. (2008) Evaluation of Project P.A.T.H.S. in Hong Kong: utilization of student weekly diary. *TheScientificWorldJOURNAL* **8**, 13–21.
18. Shek, D.T.L. and Sun, R.C.F. (2008) Implementation of a positive youth development program in a Chinese context: the role of policy, program, people, process, and place. *TheScientificWorldJOURNAL* **8**, 960–996.
19. Shek, D.T.L. and Sun, R.C.F. (2008) Implementation quality of a positive youth development program: cross-case analyses based on seven cases in Hong Kong. *TheScientificWorldJOURNAL* **8**, 1075–1087.
20. Shek, D.T.L., Ma, H.K., Lui, J.H.Y., and Lung, D.W.M. (2006) Process evaluation of the Tier 1 Program of the Project P.A.T.H.S. *TheScientificWorldJOURNAL* **6,** 2264–2273.
21. Shek, D.T.L., Ma, H.K., Sun, R.C.F., and Lung, D.W.M. (2008) Process evaluation of the Tier 1 Program (Secondary 1 Curriculum) of the Project P.A.T.H.S.: findings based on the Full Implementation Phase. *TheScientificWorldJOURNAL* **8**, 35–46.
22. Shek, D.T.L. and Sun, R.C.F. (2006) Implementation of the Tier 1 Program of the Project P.A.T.H.S.: interim evaluation findings. *TheScientificWorldJOURNAL* **6,** 2274–2284.
23. Shek, D.T.L., Ma, H.K., and Sun, R.C.F. (2008) Interim evaluation of the Tier 1 Program (Secondary 1 Curriculum) of the Project P.A.T.H.S.: first year of the Full Implementation Phase. *TheScientificWorldJOURNAL* **8**, 47–60.
24. Morgan, D.L. (1988) *Focus Groups as Qualitative Research*. Sage, Newbury Park, CA.
25. Twinn, S. (2000) The analysis of focus group data: a challenge to the rigour of qualitative research. *Nurs. Times Res.* **5(2)**, 140–146.
26. Carey, M.A. and Smith, M.W. (1994) Capturing the group effect in focus groups: a special concern in analysis. *Qual. Health Res.* **4(1)**, 123–127.
27. Albrecht, T.L., Johnson, G.M., and Walther, J.B. (1993) Understanding communication processes in focus groups. In *Successful Focus Groups: Advancing the State of the Art*. Morgan, D.L., Ed. Sage, Newbury Park, CA. pp. 51–64.
28. Peterson, A. and Esbensen, F.A. (2004) The outlook is G.R.E.A.T. What educators say about school-based prevention and the Gang Resistance Education and Training (G.R.E.A.T.) Program. *Eval. Rev.* **28(3)**, 218–245.
29. Joint Committee on Standards for Educational Evaluation (1994) *The Program Evaluation Standards*. Sage, Thousand Oaks, CA.
30. Shek, D.T.L., Tang, V., and Han, X.Y. (2005) Quality of qualitative evaluation studies in the social work literature: evidence that constitutes a wakeup call. *Res. Soc. Work Pract.* **15**,180–194.

31. Stufflebeam, D.L. (2000) The CIPP model for evaluation. In *Evaluation Models: Viewpoints on Educational and Human Services Evaluation.* Stufflebeam, D.L., Medaus, G.F., and Kellaghan, T., Eds. Kluwer Academic, Boston. pp. 279–318.
32. Shek, D.T.L., Ng, H.Y., Lam, C.W., Lam, O.B., and Yeung, K.C. (2003) *A Longitudinal Evaluation Study of a Pioneering Drug Prevention Program (Project Astro MIND) in Hong Kong*. The University of Hong Kong, Hong Kong.
